# Impact of pandemic lockdown on learning behaviour and sleep quality in German students: Results of an online survey before and during the pandemic

**DOI:** 10.1007/s11818-022-00346-8

**Published:** 2022-04-05

**Authors:** Naomi Staller, Lisa Kalbacher, Christoph Randler

**Affiliations:** Department of Biology, Eberhard Karls University, 72076 Tübingen, Germany

**Keywords:** Teleworking, Questionnaire, Social isolation, Severe acute respiratory syndrome coronavirus 2, Coronavirus disease 19, Telearbeit, Fragebogen, Soziale Isolierung, „Severe acute respiratory syndrome coronavirus type 2“, „Coronavirus disease 2019“

## Abstract

**Background:**

This study addresses the effects of the coronavirus disease 19 (COVID-19) restriction measures on sleep and bedtime habits of *N* = 637 German university students.

**Methods:**

The questionnaire was distributed online during two different time periods in 2020 (February 27–March 21) and in 2021 (February 27–March 27). The first data collection phase was immediately before the first strict lockdown to contain the COVID-19 pandemic in Germany, and the second data collection phase was during the second lockdown. The survey was composed of validated questionnaires and additional questions regarding the changes in sleep/bedtimes and the status of lectures during the lockdown phase.

**Results:**

The average Pediatric Daytime Sleepiness Scale (PDSS) score in the sample decreased during the lockdown phase, corresponding to the fact that students were less burdened with daytime sleepiness. Moreover, the sample had earlier rise and earlier bedtimes on free days during the lockdown period. Furthermore, the increase in flexible learning times brought about by the pandemic negatively impacted the students’ lifestyle and increased irregularities in sleeping habits.

**Conclusion:**

Significant changes in sleeping patterns seem to be attributable to the pandemic lockdown as found in this self-reported student survey. While daytime sleepiness decreased and earlier overall bedtimes were noted, the impact on the irregularity of sleeping and learning patterns seems to be the most notable finding, as this affects overall quality of life and learning performance. Further studies are needed to validate these findings.

## Abstract (de)

**Hintergrund:**

In dieser Studie untersuchten die Autoren die Auswirkungen und nachfolgenden Veränderungen der COVID-19-Restriktionsmaßnahmen („coronavirus disease 2019“) auf die Schlaf- und Schlafenszeitgewohnheiten von *n* = 637 deutschen Universitätsstudierenden.

**Methoden:**

Der Fragebogen wurde online zu 2 verschiedenen Zeitpunkten verteilt: 2020 (27. Februar bis 21. März) und 2021 (27. Februar bis 27. März). Die erste Datenerhebungsphase wurde unmittelbar vor der ersten strengen Abriegelung zur Eindämmung der COVID-19-Pandemie in Deutschland durchgeführt, die zweite Datenerhebungsphase fand während der zweiten Abriegelung statt. Die Erhebung setzte sich aus validierten Fragebögen und zusätzlichen Fragen zu den Veränderungen der Schlaf‑/Bettgehzeiten und dem Stand der Vorlesungen während der Abriegelungsphase zusammen.

**Ergebnisse:**

Der Durchschnittswert der Pediatric Daytime Sleepiness Scale (PDSS) in der Stichprobe sank während der Schließungsphase, was darauf hindeutet, dass die Studierenden weniger durch Tagesschläfrigkeit belastet waren. Außerdem stand die Stichprobe an freien Tagen während der Schließungsphase früher auf und ging früher ins Bett. Darüber hinaus wirkte sich die durch die Pandemie bedingte Zunahme flexibler Lernzeiten negativ auf den Lebensstil der Studierenden aus und führte zu mehr Unregelmäßigkeiten in den Schlafgewohnheiten.

**Schlussfolgerungen:**

Wie aus der Selbstauskunft der Studierenden hervorgeht, scheinen signifikante Veränderungen im Schlafverhalten auf die Pandemiesperrung zurückzuführen zu sein. Während die Tagesschläfrigkeit abnahm und insgesamt frühere Schlafenszeiten festgestellt wurden, scheint die Auswirkung auf die Unregelmäßigkeit der Schlaf- und Lerngewohnheiten das bemerkenswerteste Ergebnis zu sein, da dies die allgemeine Lebensqualität und die Lernleistung beeinflusst. Weitere Studien sind erforderlich, um diese Ergebnisse zu validieren.

With the spread of the novel coronavirus disease (COVID-19) in December 2019, a global pandemic developed, forcing governments worldwide to enforce restrictions in order to contain further dissemination. In Germany, the federal government opted for a strict lockdown, closing schools and universities, retail outlets, restaurants, sports venues, and many other economic areas. The first lockdown began on 22 March 2020, with initial economic sector easing measures coming into force on 4 May 2020. On 15 June 2020, these were introduced in almost every economic area. The summer of 2020 passed, for the most part, without rising infection figures. However, the figures rose again rapidly in autumn, first resulting in a “lockdown light” (04.11.–13.12.2020) and later in a complete lockdown (commencing 13.12.2020, with the possibility of relaxations decided in the “*Bundes-Notbremse*”—a national plan for crisis management starting 23.04.2021—with a general end to the lockdown on 30.06.2021).

The increased restriction of public life and social contact led to the population finding itself in a state of social isolation. Remote working (termed “home office” in Germany) is seen as an important factor in limiting personal contacts, which on a secondary level results in containment of transmission risks and thus of infection [1]. Therefore, the government recommended the switch to working from home for industry/business, and implemented this directly in areas under the direction of the federal and state governments (e.g., schools and universities). In accordance with these provisions, restrictions on university and school classroom teaching were imposed, which were subsequently abolished in favour of digital teaching from home. This change in lecturing (online classes ranging from fixed schedules to on-demand courses) and the additional closure of associated buildings (e.g., libraries) forced students to study almost exclusively from home. This resulted in some profound secondary changes, such as commuting time falling away and a lack of exchange with teachers and fellow students.

In this novel situation, students were confronted with subjectively perceived and physiologically measurable behavioural changes, accompanied by changes in habits such as eating habits (e.g. [2]), sleeping habits (e.g. [3, 4]) and exercise levels (e.g. [5, 6]). In this study, we investigate the relationship between sleep times on weekdays/at weekends and teaching times (measured on a scale ranging from “fixed learning times” to “on-demand learning”), as well as possible changes in social jetlag—defined as the discrepancy between the internal biological clock and the actual sleep timing due to social factors [7]—because of the pandemic. The present sample contains data from two sampling periods. The first before the lockdown and the second during the lockdown. This distinguishes the present results from those of Staller and Randler (2020) [4], as the data of their study were collected retrospectively. Furthermore, the influence of lecture status (measured on a scale ranging from “fixed learning times” to “on-demand learning”) on sleep parameters was examined here for the first time. The focus of the study was to investigate the following hypotheses: the more flexible the teaching times, the more flexible the bed- and wake-up times during the pandemic. Therefore, students live more in line with their chronotype and, as a consequence, social jetlag decreases.

## Methods

### Data collection

This study was carried out by the Department of Biology, Eberhard Karls University, Tübingen, Germany. The study sample comprises students of the University of Tübingen. The questionnaire was provided in German language only and distributed online during two different time periods in 2020 (February 27–March 21) and 2021 (February 27–March 27). The first data collection phase was directly before the first strict lockdown to contain the COVID-19 pandemic in Germany, and the second data collection phase was during the second lockdown. Participants were made aware of the study via mailing lists and official university platforms. The background of the study was clarified, and the voluntary and anonymous nature of participation was pointed out up front. In addition, the participants were made aware that they would not suffer any disadvantages if they terminated the survey prematurely and that participation would not be remunerated. The survey was hosted on the SoSciSurvey online platform, which fulfils the European Union’s data privacy standards. Data were collected from a total of *N* = 637 (male: 191; female: 445; other: 8) participants; first data collection *N* = 312, second data collection *N* = 325.

### Questionnaires

In addition to collecting data on demographics and the highest educational qualification (type, year and state in which the qualification was obtained), the survey was compiled from validated questionnaires (Morningness–Eveningness Stability Scale improved, MESSi; Pittsburgh Sleep Quality Index, PSQI; Sleep timing questionnaire, STQ; Pediatric Daytime Sleepiness Scale, PDSS). Furthermore, the participants provided information on the current status of lectures “type of teaching currently offered” twice, but coded slightly differently in each case, to form a scale (1: If you worked remotely from home in the past 2 months, how often did this follow a fixed schedule with set times? Exclusively/a lot/medium/little/not at all; 2: If you worked remotely from home in the past 2 months, how often was this based to on-demand events? Exclusively/a lot/medium/little/not at all). Also, participants were asked whether they had completed the survey at both survey times. Participants for whom this circumstance applied were excluded from the analysis (*N* = 398). The reasoning was that the survey took place anonymously and the datasets of this linked sample could not have been combined as dependent datasets. Therefore, independent datasets were analysed. The questionnaire took an average of 11.29 min to complete with a standard deviation of 4.76 min.

### Morningness–Eveningness Stability Scale improved

In this study we used the MESSi to assess participants’ morningness–eveningness. The questionnaire is separated into three subscales—the morning affect subscale (MA), the eveningness subscale (EV) and the distinctness subscale (DI). Each subscale consists of five questions in a 1–5 Likert format. The MA queries the affective facet (e.g., “How alert do you feel during the first half hour after awakening in the morning?”), the EV collects data on the overall physical and mental situation in the evening (e.g., “In general, how is your energy level in the evening?”), while DI is looking into the amplitude of active phases (e.g., “There are moments during the day where I feel unable to do anything.”). Higher values on the subscales indicate participants being more prone to those facets (MA → prone to morningness/EV → prone to eveningness/DI → higher daytime fluctuations of active phases). The MESSi questionnaire has been validated in various studies (factorial invariance, structure, reliability in different languages, e.g., [8–12]; and by actigraphy [13]). Cronbach’s α in the current sample was 0.891 for MA, 0.870 for EV and 0.759 for DI.

### Pittsburgh Sleep Quality Index

The PSQI is a self-reported inventory that measures various sleep variables in a retrospective design considering the past 4 weeks. It is used in clinical and non-clinical settings and includes 19 self-rated and 5 externally rated questions. The latter are not included in the quantitative evaluation. The PSQI is divided into seven sections: subjective sleep quality, sleep latency, sleep duration, habitual sleep efficiency, sleep disturbances, use of sleeping medications and daytime dysfunction over the past month (e.g., “During the past month, how often have you had trouble sleeping because you cannot get to sleep within 30 min?”). The sum of the 1–4 Likert scale leads to a classification from “good” sleepers to “poor” sleepers [14]. The current study investigated the frequency of sleep disturbances (eight items) and performed an assessment of overall sleep quality (1 item). Cronbach’s α in the current sample was 0.642.

### Sleep timing questionnaire

The STQ consists of 18 items asking for the individual bed and awakening times on weekdays and at weekends, the stability of these schedules, and the frequency and length of night awakenings. It was developed to provide a precise picture of a person’s typical sleep rhythm [15]. Sleep technicians can thus adjust the timing of polysomnographic examinations to the specific needs and habits of the patient [16]. It is more time efficient than a sleep diary format. As we had already collected sleep quality aspects, we only asked for variation individual bed and awakening times on weekdays or at weekends and, in addition, calculated the length of sleep. Cronbach’s α in the current sample was 0.831.

### Paediatric Daytime Sleepiness Scale

The PDSS is a questionnaire originally introduced in English [17] and translated into German by Schneider and Randler (2009) [18]. It queries the daytime sleepiness of students. The questionnaire contains eight items of which two are clearly related to a school or learning environment. All questions are asked in a 1–5 Likert format, with one question being reverse coded. The total score of the questionnaire is obtained by adding up the answer scores. Cronbach’s α of this scale was 0.808 in this sample.

### Habitual sleep–wake variables

We collected bedtimes and rise times on weekdays and free days to calculate sleep duration, midpoint of sleep and social jetlag. Sleep duration (SD) results from the difference between sleeping and waking times and is summed up to average sleep duration (SD_*average*_) as follows:

$$SD_{\text{average}}=\frac{5xSD_{\text{workdays}}+2xSD_{\text{freedays}}}{7}$$

The term midpoint of sleep (MS) refers to the clock time-based midpoint between sleep onset and awakening. Since bedtime and waking times differ measurably between weekdays and weekend days/days off, the term social jetlag was coined [7]. Social jetlag quantifies the difference between the midpoint of sleep on weekdays compared to the midpoint of sleep on days off. Accordingly, we use an algorithm to correct the midpoint of sleep (MS_*freedays corrected*_) and to include the weekend oversleep in the analysis [19]:

$$MS_{\text{freedays}\,\text{corrected}}=MS_{\text{freedays}}-0.5x\left(\frac{\begin{array}{c}SD_{\text{freedays}}-(5xSD_{\text{workdays}}\\+2xSD_{\text{freedays}})\end{array}}{7}\right)$$

### Statistics

Data analysis was performed using SPSS 27.0 (IBM, Armonk, NY, USA). Examined characteristics were “status of lectures”, MESSi, STQ, PDSS, PSQI and self-reported sleep variables. As not all participants filled in all questions, the exact sample size is always given for the calculations; thus, *N* and degrees of freedom may differ.

## Results

Descriptive statistics are presented in Table 1.

**Table 1:** Descriptive statistics of the study sample. Only independent datasets were analysed, which is why *N* = 398 datasets were excluded from the analysis

	*N*	Mean	Standard deviation
Morning affect	637	2.959	0.996
Eveningness	637	3.146	1.016
Distinctness	637	3.660	0.842
PSQI score	637	0.590	0.400
PDSS score	632	3.130	0.553
STQ score	632	4.679	2.042
Rise times weekdays	622	7:23	1:06
Rise times free days	575	9:16	1:34
Bedtime weekdays	608	23:11	1:12
Bedtime free days	618	00:21	1:31
Midpoint of sleep weekdays	599	3:17	0:59
Midpoint of sleep free days	572	4:49	1:27
Midpoint of sleep corrected	560	4:34	1:22
Sleep duration week	599	8:12	1:07
Sleep duration free days	572	8:54	1:04
Average sleep duration	560	8:23	0:57
Social jetlag	560	1:31	1:01

*PSQI *Pittsburgh Sleep Quality Index, *PDSS *Paediatric Daytime Sleepiness Scale, *STQ *Sleep timing questionnaire

We carried out *t*-tests to assess the influence of measurement timepoint (before vs. during the pandemic). These tests showed an influence on sleep duration on weekdays, average sleep duration, midpoint of sleep on free days, social jetlag, and PDSS scores (Table 2). Further, differences existed in rise times, and in free day bedtimes (Table 2).

**Table 2:** *T*-test for independent groups (before vs. during the pandemic) assessing the influence of timepoint on chronotype and sleep variables

Pandemic		*N*	Mean	Standard deviation	Standard error of the mean	T	Df	*P*-value	Cohen’s d
Morning affect	Before	312	2.885	1.006	0.057	−1.833	635	0.067	−0.145
	During	325	3.030	0.983	0.055	–			–
Eveningness	Before	312	3.190	0.997	0.056	1.081	635	0.280	0.086
	During	325	3.103	1.033	0.057	–			–
Distinctness	Before	312	3.724	0.834	0.047	1.887	635	0.060	0.150
	During	325	3.599	0.847	0.047	–			–
PSQI score	Before	312	0.574	0.383	0.022	−0.973	635	0.331	−0.077
	During	325	0.605	0.415	0.023	–			–
PDSS score	Before	312	3.075	0.586	0.033	−2.505	630	0.012*	−0.199
	During	320	3.184	0.514	0.029	–			–
STQ score	Before	312	4.763	1.969	0.111	1.021	630	0.307	0.081
	During	320	4.597	2.111	0.118	–			–
Rise times weekdays	Before	306	7:15	1:02	0:03	−2.941	620	0.003**	−0.236
	During	316	7:30	1:08	0:03	–			–
Rise times free days	Before	280	9:28	1:36	0:05	3.038	573	0.002**	0.253
	During	295	9:04	1:31	0:05	–			–
Bedtime weekdays	Before	297	23:14	1:11	0:04	0.995	606	0.320	0.081
	During	311	23:08	1:13	0:04	–			–
Bedtime free days	Before	300	24:30	1:29	0:05	2.494	616	0.013*	0.201
	During	318	24:12	1:32	0:05	–			–
Midpoint of sleep weekdays	Before	294	3:14	0:57	0:03	−1.254	597	0.210	−0.103
	During	305	3:20	1:02	0:03	–			–
Midpoint of sleep free days	Before	279	5:00	1:27	0:05	2.936	570	0.003**	0.246
	During	293	4:39	1:26	0:05	–			–
Midpoint of sleep corrected	Before	274	4:40	1:20	0:04	1.798	558	0.073	0.152
	During	286	4:28	1:24	0:05	–			–
Sleep duration week	Before	294	8:01	1:07	0:03	−3.863	597	<0.001***	−0.315
	During	305	8:22	1:05	0:03	–			–
Sleep duration free days	Before	279	8:56	1:05	0:03	0.644	570	0.520	0.054
	During	293	8:52	1:03	0:03	–			–
Average sleep duration	Before	274	8:16	0:55	0:03	−3.036	558	0.003**	−0.257
	During	286	8:30	0:58	0:03	–			–
Social jetlag	Before	274	1:45	1:03	0:03	5.537	558	<0.001***	0.467
	During	286	1:17	0:55	0:03	–			–

*Df* degrees of freedom, *PSQI* Pittsburgh Sleep Quality Index, *PDSS* Pediatric Daytime Sleepiness Scale, *STQ* Sleep timing questionnaire

*p < 0.05, **p < 0.01, ***p < 0.001

Furthermore, we assessed the relationship of the type of instruction offered to students during the pandemic with chronotype and sleep variables. In the questionnaire, the participants answered the question “type of teaching currently offered (status of lectures)” twice, but the questions were reverse coded to each other. We linked these two variables via an exploratory factor analysis (principal component, varimax rotation) with the residuals saved as factor scores (regression factor 1, Table 3). “Fixed schedule” loaded positively while “on-demand events” loaded negatively on the scale. A high positive value of the residuals (regression factor) corresponds to a flexible schedule.

**Table 3:** Spearman’s rho rank-order correlation coefficients for the interaction of the variables “fixed lecture time” and “on-demand lectures” with chronotype and sleep variables

Spearman’s rho		Regression factor
Morning affect	Correlation coefficient	−0.111
	Significance (two-tailed)	0.066
	*N*	276
Eveningness	Correlation coefficient	0.074
	Significance (two-tailed)	0.222
	*N*	276
Distinctness	Correlation coefficient	0.025
	Significance (two-tailed)	0.678
	*N*	276
PSQI score	Correlation coefficient	−0.058
	Significance (two-tailed)	0.336
	*N*	276
PDSS score	Correlation coefficient	−0.112
	Significance (two-tailed)	0.064
	*N*	272
STQ score	Correlation coefficient	0.145*
	Significance (two-tailed)	0.017*
	*N*	271
Rise time weekdays	Correlation coefficient	0.160**
	Significance (two-tailed)	0.008**
	*N*	269
Rise time free days	Correlation coefficient	0.156*
	Significance (two-tailed)	0.013*
	*N*	252
Bedtime weekdays	Correlation coefficient	0.165**
	Significance (two-tailed)	0.007**
	*N*	266
Bedtime free days	Correlation coefficient	0.172**
	Significance (two-tailed)	0.005**
	*N*	271
Midpoint of sleep weekdays	Correlation coefficient	0.182**
	Significance (two-tailed)	0.003**
	*N*	261
Midpoint of sleep free days	Correlation coefficient	0.181**
	Significance (two-tailed)	0.004**
	*N*	251
Midpoint of sleep corrected	Correlation coefficient	0.171**
	Significance (two-tailed)	0.007**
	*N*	245
Sleep duration weekdays	Correlation coefficient	−0.030
	Significance (two-tailed)	0.631
	*N*	261
Sleep duration free days	Correlation coefficient	−0.094
	Significance (two-tailed)	0.137
	*N*	251
Average sleep duration	Correlation coefficient	−0.028
	Significance (two-tailed)	0.659
	*N*	245
Social jetlag	Correlation coefficient	0.032
	Significance (two-tailed)	0.620
	*N*	245

*PSQI *Pittsburgh Sleep Quality Index, *PDSS *Paediatric Daytime Sleepiness Scale, *STQ *Sleep timing questionnaire

*p < 0.05, **p < 0.01, ***p < 0.001

## Discussion

The aim of this study was to investigate the changes in sleeping and learning behaviour attributable to the pandemic lockdown. There were three main findings that can be summarized as follows:

1. The average PDSS score in the sample decreased during the lockdown phase, corresponding to the fact that students were less burdened with daytime sleepiness.
2. On free days, students had earlier rising times and earlier bedtimes during the lockdown period.
3. The increase in flexible learning times brought on by the pandemic negatively impacted students’ lifestyle and increased irregularities in sleeping habits.

The decrease in the average PDSS score during the lockdown period is in line with the results of the rise times during the week. Our data demonstrate that students rose later by an average of 15 min on weekdays during the lockdown period. Taken together with the trend toward earlier rise times on free days (on average 24 min earlier compared to the pre-lockdown period), this indicates that the students were able to align their schedules more with their internal biological rhythm. In sum, this led to a decline in social jetlag by 28 min. Students increased their sleep duration during the week by 21 min on average. Their average sleep duration (weekdays + free days) increased by 14 min. Similar results in the investigation of altered sleep timing were also shown in, e.g., Italian [3], another German [4], North American [20, 21], Chinese [22] and Singaporean [23] cohorts. For example, Ong et al. (2021) [23] reported a reduction in weekday–weekend difference in social jetlag of 50 min. Accordingly, the lockdown and the subsequent changes in getting up times attributable to (1) the flexible teaching schedule and (2) the elimination of commuting times enabled the majority of students to reduce their sleep deficit during the week. Therefore, students may have already been well-rested at an earlier time during the weekend and, subsequently, rose earlier without the need to compensate for the weekday sleep deficit. This is further strengthened by the results of a large US survey on sleep times and their relationship to waking activities. Here, it was shown that the second largest reciprocal relationship to sleep (after work time) was found for travel time including commuting times [24] (for conflicting results and an alternate interpretation see Putilov, 2021; [25]).

In addition to earlier rise times on free days, students also had later bedtimes (on average by 18 min), which complements the results of the midpoint of sleep on free days (on average 21 min later) well. Several explanations which fit into the chain of reasoning are possible. For example, the earlier bedtime and the earlier midpoint of sleep on weekends may be due to the fact that leisure activities were severely restricted during the lockdown. In some areas, people were no longer allowed to leave their homes in the evening due to strict curfews. Moreover, severe contact restrictions were enforced, which is why the reception of visitors also declined sharply. The significant decline in leisure opportunities and the general stay-at-home attitude may have prompted the shift in bedtimes during the lockdown. These results advocate a state of social isolation in the sample group [26]. A less likely explanation would be that students spent less time commuting during the pandemic, leaving more time for personal leisure (if working hours remained the same), conveying the impression of an improved work–life balance. In consequence, they might have been more likely to rest more in themselves and able to go to bed earlier, including at the weekends. The exact reasons remain speculative and would need to be verified by further studies on the psychological effects of lockdown.

The evaluation of the status of lectures in the lockdown subsample showed effects on all clock time-dependant sleep parameters. The results of the factor analysis suggested that the subjects’ lifestyle regularity changed negatively due to flexible learning times. The results support the hypothesis that if the subjects were able to choose their own times for getting up and going to bed due to flexible learning times, the irregularity in their sleep timing would increase. This may negatively impact sleep rhythm and has been associated with serious health issues in the long-term course [27]. Work itself is a strong frame for daytime organization (see also [23]). This reflects the disadvantages of flexible learning times. However, in contrast to fixed learning times (which tend to stabilise lifestyle regularity), flexible learning times give subjects the opportunity to adapt their sleep and waking times to their own biological rhythm and thus reduce social jetlag. Taken together, our results show an increase in lifestyle irregularity, which has a negative effect on sleep timing on one hand, and a positive effect on an individual’s personal sleep rhythm and sleep timing due to a reduction of social jetlag on the other. The data on the PSQI score prior to and during the pandemic did not change significantly, showing that even though the lockdown influenced sleep-dependent variables, participants reported no effect on their sleep quality or the amount of sleep disturbances. A possible explanation for this result is that these opposing influencing factors (increased lifestyle irregularity/decreased social jetlag) cancel each other out. However, the method used here to assess sleep quality (facet on sleep quality of the PSQi score) consists only of a single item that is also self-reported (vs. e.g., physiological measurements). To confirm the discussed effect, further studies with a broader spectrum of survey methods for sleep quality should be connected.

These results show that the consequences of the lockdown must be considered in a multilayered manner. In principle, more flexible lecture times help students to organise their everyday life to be more in line with their own chronotype. This leads to cultivating a healthier sleep and less suffering from the consequences of social jetlag, such as depression [28]. However, during the lockdown, not only did the university environment have to be restructured with immense restrictions, but also all other facets of everyday life. The students thus lost a large part of the structure and regularity in their everyday lives. A higher irregularity in life, however, leads to poorer sleep due to a poorer sleep rhythm. We conclude that the most health-promoting version of the lecture offer may be a hybrid model combining fixed lecture times, where questions can be clarified, and an on-demand offer.

## Strengths and limitations

The strengths of this research are that the data were collected during the actual experience before or during the lockdown and the subjects did not have to provide retrospective assessments. Limitations are mainly the self-assessment questionnaire design of the study, which was not backed up by peer assessment or physiological data.

Since the anonymity of the participants of this study was preserved, we did not encode the participants, so we could not precisely track individual changes in sleep habits before and during the pandemic.

## Conclusion

Significant changes in sleeping patterns seem to be attributable to the pandemic lockdown, as found in this self-reported student survey. While daytime sleepiness decreased and earlier overall bedtimes were noted, the impact on the irregularity of sleeping and learning patterns seems to be the most notable finding, as this affects overall quality of life and learning performance. Further studies are needed to validate these findings.

## Declarations

The data basis of the present study is an online ques onnaire that was collected completely anonymously, no personal data was requested and there was no physical or mental stress or decep on, which is why no ethics approval needs to be obtained under German law.
